# Two new species of *Lithobius* on Qinghai-Tibetan plateau identified from morphology and COI sequences (Lithobiomorpha: Lithobiidae)

**DOI:** 10.3897/zookeys.785.28580

**Published:** 2018-09-13

**Authors:** Penghai Qiao, Wen Qin, Huiqin Ma, Tongzuo Zhang, Jianping Su, Gonghua Lin

**Affiliations:** 1 Key Laboratory of Adaptation and Evolution of Plateau Biota, Northwest Institute of Plateau Biology, Chinese Academy of Sciences, Xining 810008. No.23 Xinning Road, Chengxi District, Xining, Qinghai, China Northwest Institute of Plateau Biology, Chinese Academy of Sciences Xining China; 2 Qinghai Provincial Key Laboratory of Animal Ecological Genomics, Xining, Qinghai, China Qinghai Provincial Key Laboratory of Animal Ecological Genomics Xining China; 3 Graduate University of the Chinese Academy of Sciences, Beijing 100049, China Graduate University of the Chinese Academy of Sciences Beijing China; 4 Scientiﬁc Research Oﬃce, Hengshui University, Hengshui 053000, China Hengshui University Hengshui China

**Keywords:** China, COI, *
Ezembius
*, Lithobiidae, Qinghai-Tibet Plateau, taxonomy

## Abstract

Lithobius (Ezembius) longibasitarsus**sp. n.** and Lithobius (Ezembius) datongensis**sp. n.** (Lithobiomorpha: Lithobiidae), recently discovered from Qinghai-Tibet Plateau, China, are described. A key to the species of the subgenus Ezembius in China is presented. The partial mitochondrial cytochrome *c* oxidase subunit I barcoding gene was amplified and sequenced for eight individuals of the two new species and the dataset was used for molecular phylogenetic analysis and genetic distance determination. Both morphology and molecular data show that the specimens examined should be referred to Lithobius (Ezembius).

## Introduction

The myriapod fauna of China has been poorly investigated and this is especially the case with centipedes of the order Lithobiomorpha, with only approximately 80 species/subspecies of lithobiomorphs are known from the country. Qinghai-Tibet Plateau is among the very poorly studied regions of China (Ma et al. 2014a, b, 2015, 2018, Pei et al. 2014, 2015, 2016, 2018, Qiao et al. 2018, Qin et al. 2014, 2017). Altogether, 20 species of Lithobius (Ezembius) have been recorded from China, but none of them have been reported from Qinghai Province (Pei et al. 2018). Herein Lithobius (Ezembius) longibasitarsus sp. n. and Lithobius (Ezembius) datongensis sp. n. are described and illustrated, both from Qinghai Province.

The centipede subgenus Ezembius was erected by Chamberlin (1919) as a monotypic genus to receive *Lithobiusstejnegeri* Bollman, 1893 from Bering Island and was then formally proposed as new and described by Chamberlin (1923). It accommodates a group of 58 species/subspecies known mostly from Asia, but also western North America and spans a wide range of habitats from the arctic and sub-arctic to tropical and sub-tropical forests, to steppe and overgrazed stony areas of central Asia, and Himalayan montane forests, from the sea shore up to 5500 m (Himalayas) (Zapparoli and Edgecombe 2011). *Ezembius* is characterized by antennae with ca. 20 articles, ocelli 1+4–1+20, forcipular coxosternal teeth usually 2+2, porodonts generally setiform but sometimes stout, tergites generally without posterior triangular projections, tarsal articulation of legs 1–13 distinct, female gonopods with uni-, bi- or tridentate claw, 2+2–3+3, rarely 4+4 spurs (Zapparoli and Edgecombe 2011).

## Material and methods

*Specimen collection and preparation*: the specimens were all collected by hand, preserved in 95% ethanol, and deposited in the collections of Northwest Institute of Plateau Biology (**NWIPB**), Chinese Academy of Sciences. Characters were examined using an Olympus SZ61 stereoscope. Terminology for external anatomy follows Bonato et al. (2010). Specimens are numbered from 1 to 12 according to collection quantity and prefixed with the abbreviation of the locality. Abbreviations used in the text are as follows:

**a** anterior;

**C** coxa;

**D** dorsal;

**DT** Datong.

**F** femur;

**GH** Gonghe,

**m** median;

**P** prefemur;

**p** posterior;

**T, TT** tergite, tergites;

**S, SS** sternite, sternites;

**Ti** tibia;

**To** Tömösváry’s organ;

**Tr** trochanter;

**Ts I** tarsus I;

**Ts II** tarsus II;

**V** ventral.

*DNA extraction and sequencing protocols*: standard DNA extraction and amplification methods were performed. Total DNA was extracted from a single leg removed from each specimen sample using MicroElute Genomic DNA kit (OMEGA), after overnight incubation at 65°C. Polymerase chain reactions (PCRs) were conducted using Mastercycler pros PCR (Eppendorff) in total reaction volumes of 39μL volumes containing 5–60 ng template DNA, 1μL; ddH2O 28μL; 10×Buffer 5μL (Takara, Dalian, China); 0.5mm/L dNTPs 2.5μL (Takara, Dalian, China); 5U/μL Taq polymerase 0.5μL (Takara, Dalian, China); Forward Primer 1μL; Reverse Primer 1μL (synthesized by Sangon Biotech from Shanghai). A 686 bp fragment of COI was amplified using the primers LCO1490/LCO2198 (Edgecombe et al. 2002). PCR was performed as follows: initial denaturing at 95°C for 10 min; followed by 35 cycles of 95°C for 30 s, 44°C for 30 s, and 72°C for 90 s and a final extension at 72°C for 10 min. The PCR products were purified using a purification kit (DC28106 250 Preps, QIAGEN, Germany). Sequencing reactions were implemented using ABI Prism BigdyeTM Terminator Cycle Sequencing Ready Reaction Kit on ABI 3730XL sequencer, with the PCR primers.

The GenBank accession numbers of all eight new sequences were MH05602–MH045609 (*Ezembius* COI). Sequence identities were confirmed with BLAST searches (Altschul et al. 1997). In order to eliminate indicators of nuclear mitochondrial pseudogenes (numts), such as indels, stop codons and double peaks in sequence chromatograms, the whole dataset was translated into amino acids using the ‘invertebrate’ code in MEGA6 (Tamura et al. 2013); internal stop codons were absent in our dataset; gaps were absent.

*Phylogenetic analyses*: the sequences were aligned with Clustal X2.0 (Chenna et al. 2003). The aligned sequences were edited using the program BioEdit 7.0.9.0 (Hall 1999) by hand. The substitution model selection was implemented in jModelTest 2.1.4 (Darriba et al. 2012), the TIM3+I+G model was selected by likelihood ratio tests under the Akaike Information Criterion (AIC 11833.1212) and the Trn+I+G model under the Bayesian Information Criterion (BIC 12085.5234). Topology was reconstructed under the Trn+I+G model of nucleotide evolution in MrBayes. Bayesian inference (BI) was used to generate a phylogenetic hypothesis of the DNA haplotypes. BI was performed in MrBayes version 3.1.2 (Ronquist and Huelsenbeck 2003) with 3,000,000 generations, sampling trees every 300 generations. Two independent runs each with four simultaneous Monte Carlo Markov chains (MCMC) were carried out. The first 25% of generations were discarded as ‘burn-in’. The convergence of chains was confirmed until average standard deviation of split frequency is below 0.01 (0.002825) and the potential scale reduction factor (PSRF) is close to 1.0 for all parameters. In phylogenetic analysis *Anopsobiusneozelanicus* Silvestri, 1909 was used as outgroup.

*Distance analysis*: the analysis involved 26 nucleotide sequences (App. 1). Codon positions included were 1^st^+2^nd^+3^rd^. All ambiguous positions were removed for each sequence pair. There were a total of 613 positions in the final dataset. Evolutionary analyses were conducted in MEGA6 (Tamura et al. 2013). All pair-wise intra- and inter-specific distances were produced to evaluate species divergence in *Ezembius*.

## Taxonomic accounts

### Class Chilopoda Latreille, 1817

#### Order Lithobiomorpha Pocock, 1895

##### Family Lithobiidae Newport, 1844

###### Subfamily Lithobiinae Newport, 1844

####### Genus *Lithobius* Leach, 1814

######## Subgenus Ezembius Chamberlin, 1919

######### Lithobius (Ezembius) longibasitarsus sp. n.

Taxon classificationAnimaliaLithobiomorphaLithobiidae

########## Type material.

**Figure 1.**
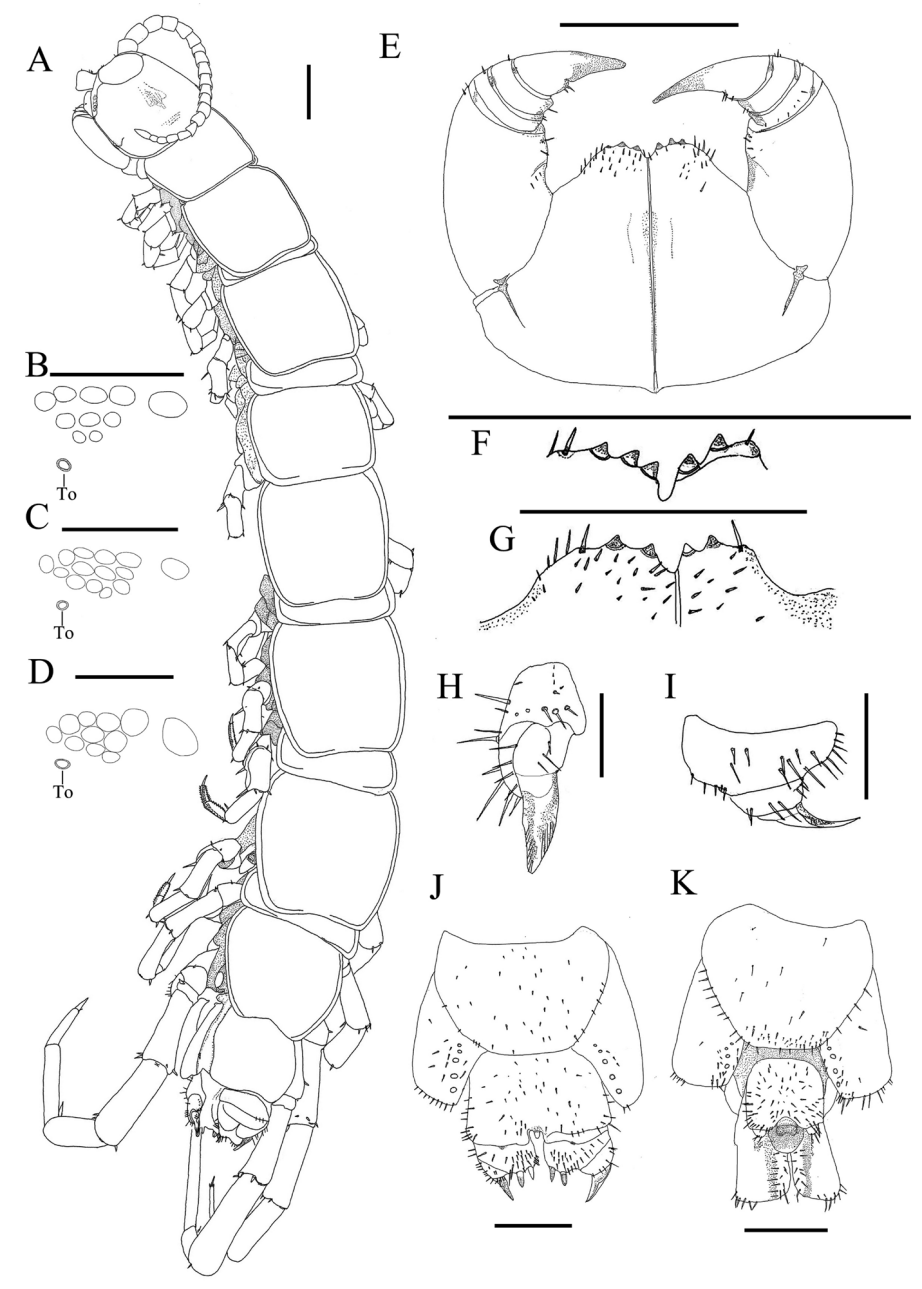
Lithobius (Ezembius) longibasitarsus sp. n., **A, D, E, H–J** holotype, female: **A** dorsal view **D** ocelli and To, lateral view **E** forcipular coxosternite, ventral view **H** posterior segments and gonopods, dorsal view **I** claw of female gonopod, inboard view **J** female posterior segments and gonopods, ventral view **B, F** paratype, female, labelled GH4: **B** ocelli and To, lateral view **F** forcipular coxosternite, ventral view **C, G, K** paratype, male, labelled GH8: **C** ocelli and To, lateral view **G** forcipular coxosternite, ventral view **K** posterior segments and gonopods, ventral view. Scale bars 1 mm **A, E, F, G**; 300 μm **B, C, D, H, I**; 500 μm **J**.

Holotype: female labelled GH3 (Figure 1 A, D–E, H–G), body length 17.0 mm, from Gonghe County, Qinghai province, China, 36.64508° N 100.80747° E, 14 July 2011, 3287 meters above sea level, collected by Gonghua Lin. Paratypes: one female, one male, same data as holotype.

########## Habitat.

Specimens were collected under stones on steppes covered with legume shrubs and grass composed mainly of Poaceae. The sampling point belongs to the Gonghe Basin region of the Tibet plateau severely affected by desertification.

########## Etymology.

The specific name refers to the new species with a long tarsus I of leg XV, tarsus I approx. 1.7 times longer than tarsus II.

########## Diagnosis.

Body length 17.0–18.0 mm; head slightly widened; antennae of 20 articles; 10–14 ocelli arranged in three irregular rows; To oval to round, slightly smaller in size to neighbouring ocelli; lateral margins of forcipular coxosternite slanting; anterior margin with 2+2, 3+2 or 2+3 blunt teeth and with strong setiform porodonts; tergites without triangular posterolateral process; legs XIV and XV thicker and longer than anterior ones in both sexes; coxal pores 4–6, round to ovate arranged in one row; female gonopods with two moderately long, bullet-shaped spurs; terminal claw of the third article simple, with a small triangular protuberance on basal ventral side; male gonopods short and small.

########## Description.

Holotype (♀), body 17.0 mm long, cephalic plate width 2.1 mm, length 2.0 mm.

*Colour*: antennae light yellow; tergites pale yellow-brown; cephalic plate and terminal tergite yellow-brown; pleural region and sternites pale yellow; distal part of forcipules dark brown, maxillipede coxosternum and SXV yellow; legs pale yellow with grey hue, pretarsal claw brown.

*Antennae* composed of 20+20 articles (Figure 1 A), length 3.31 mm, basal article slightly wider than long, second article with equal length and width, the following articles longer than wide, distal article 2.6 times as long as wide; abundant setae on antennal surface.

*Ocelli area* translucent with dark pigment, 1+5, 3, 2 ocelli on each side of cephalic plate, arranged in three irregular rows. The posterior ocellus is the biggest. To oval, smaller than the adjacent ocelli, situated ventrally on anterolateral margin of cephalic plate.

*Cephalic plate* smooth, slightly broader than long; as broad as TIII or slightly broader. Frontal marginal of head with clear transverse suture. Posterior margin slightly concave; projection of lateral marginal conspicuously discontinuous; posterior marginal ridge slightly concave with median thickening.

*Coxosternite* subtrapezoidal, anterior margin narrow, lateral margins of the coxosternite slightly longer than medial margins. Median diastema shallow, U-shaped; anterior margin with 3+2 blunt nipple-like teeth (Figure 1E). Porodonts thick and strong separated from the lateral tooth ventrolaterally. Scattered short setae on the ventral side of coxosternite, longer setae near the dental margin and the porodonts.

*Tergites* all smooth, without wrinkles, TI narrower posterolaterally than anterolaterally, generally trapezoidal, narrower than the cephalic plate and TIII, the cephalic plate almost the same width as TIII. Posterior marginal ridge of TI straight; of TT III, V shallow concave; of TT VIII, X, XII slightly concave; of TXIV deeply concave; TT VI– XIV bordered laterally only (Figure 1A). Posterior angles of all tergites rounded without triangular projections. Only one or two pairs of setae on anterior angles of each tergite.

*Sternites*: posterior part of sternites narrower than anterior, generally trapezoidal, smooth; 2–8 setae on anterior angle, anterior lateral side, posterior angle and posterior lateral side; some minute setae on SS XIV and XV, most of which distributed on posterior lateral margins and posterior borders.

*Legs*: tarsal articulation well defined on legs I-XV. All legs with fairly long curved claws. Legs I–XIV with anterior and posterior accessory spurs, anterior accessory spur moderately long and slender ca. 33%-50% the length of principle claw, the posterior one stouter forming slightly larger angles with tarsal claws, ca. 0.25 the length of principal claw. Legs XV lacking anterior and posterior accessory spurs. Dense glandular pores on the surface of prefemur, femur, tibia, and tarsi of legs XIV and XV. Short to long setae sparsely scattered over the surface of prefemur, femur, tibia, and tarsi of legs I-XIII, more setae on the tarsal surface, with two rows of comb-like setae along ventral side, fewer setae on legs XIV and XV. Legs XIV and XV moderately thicker and longer than anterior legs, tarsus I ca. 6.6 times as long as wide, tarsus II ca. 37% length of the whole tarsus on leg XV. Leg plectrotaxy as presented in Table 1.

*Coxal pores* circular on legs XII–XV, separated by a distance 1–2 times larger than diameter of pore; inner pores smaller; formula 6, 5, 5, 5. Coxal pores set in a shallow groove arranged in a row with short to long setae scattered over the surface of apophysis (Figure 1J).

*Female posterior segment*: S XV generally trapeziform, straight posteromedially; sternite of genital segment wider than long with posterior margin moderately concave between condyles of gonopods, except for a small, median bulge; distal part lightly sclerotised; short to long setae scattered over the surface of genital segment and lateral margins. The first article of gonopod moderately broad bearing 22–24 short to moderately long setae arranged in three rows with 2+2 moderately long, bullet-shaped spurs, inner spur slightly smaller and more anterior than the outer (Figure 1J), four short setae, and three long setae on dorsolateral ridge (Figure 1H). The second article of gonopod with 8–10 setae, three long setae along the dorsolateral ridge (Figure 1H). Third article of gonopod with six moderately long setae. Terminal claw simple, slender and sharp, having small triangular protuberance on ventral side (Figure 1I).

*Male posterior segment*: S XV subtrapeziform, long setae scattered sparsely over its surface and posterior margins. Male genital sternite slightly wider than long; posterior margin quite deeply concave between the gonopods, no bulge medially; ca. 69 short to medium setae scattered sparsely over its surface and at lateral margins; gonopods of a single small semicircular article with 3-5 seta on its surface (Figure 1K). Male leg XV not modified.

**Table 1.** Lithobius (Ezembius) longibasitarsus sp. n.: leg plectrotaxy; letters in brackets indicate variable spines.

legs	ventral					dorsal				
	C	Tr	P	F	Ti	C	Tr	P	F	Ti
1			(a)mp	amp	am			ap	ap	ap
2–3			amp	amp	am			ap	ap	ap
4–7			amp	amp	am			a(m)p	ap	ap
8–11			amp	amp	am			amp	ap	ap
12			amp	amp	am	(a)		amp	ap	ap
13			amp	amp	am	a		amp	p	ap
14		m	amp	amp	am	a		amp	p	p
15		m	amp	am	a	a		amp	p	(p)

########## Variations.

Body length 17.0–18.0 mm; ocelli 1+5, 4, 4 or 1+5, 3, 2 or 1+4, 3, 2 (Figure 1 B–D); coxal pores 5544, 5554 or 6555; coxosternal teeth 2+2, 3+2 or 2+3 (Figure 1 E–G).

########## Remarks.

Lithobius (E.) longibasitarsus sp. n. can be distinguished from all the other known Chinese species of subgenus Ezembius Chamberlin, 1919 by 2+2, 2+3 or 3+2 moderately blunt teeth on the forcipular coxosternite and the terminal claw of the female gonopod simple, slender and sharp, having a small triangular protuberance on its ventral side. It has a larger body (17.7–18.0 mm), more ocelli (10–14), more coxal pores (5544, 5554 or 6555), and DaC spine on legs XII and XV.

Morphologically it resembles L. (E.) tetraspinus but can be readily distinguished by the following characters: more ocelli (10–14 vs. 9–10), more coxosternal teeth (2+3, 3+2 vs. 2+2), more coxal pores (4–6 vs. 2–5), and less spurs on female gonopods (2+2 contrary to 2+3 or 3+2).

######### Lithobius (Ezembius) datongensis sp. n.

Taxon classificationAnimaliaLithobiomorphaLithobiidae

http://zoobank.org/05A00271-9A67-4226-87DD-2BDB240AE1FF

########## Type material.

**Figure 2.**
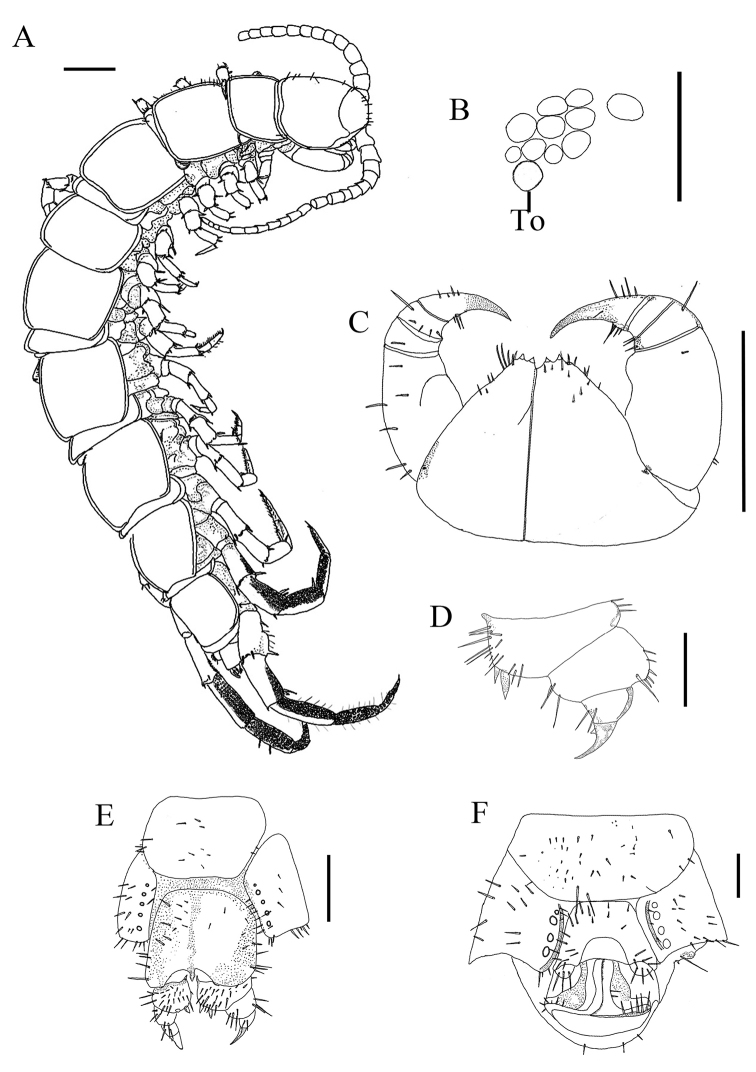
Lithobius (Ezembius) datongensis sp. n., **A, C–E** holotype, female: **A** habitus, dorsal view; **C** forcipular coxosternite, ventral view **D** female gonopods, dorsal lateral view **E** female posterior segments and gonopods **B, F** paratuype, male: **B** ocelli and Tömösváry’s organ (To), lateral view **F** posterior segments and gonopods, ventral view. Scale bars 1 mm **A, C**; 300 μm **B, D**; 500 μm **E, F.**

Holotype: female labelled DT5 (Figure 2), body length 14.2 mm, from Datong County, Qinghai province, China, 37.12494° N 101.811611° E, 21 October 2010, 2950 meters above sea level, collected by Gonghua Lin. Paratypes: one female, one male, same data as holotype.

########## Habitat.

Specimens were collected under stones of slope-lands covered with grass mainly of *Pedicularischinensis* and shrub mainly of *Potentillafruticosa* along the riverside in coniferous forest composed mainly of *Piceacrassifolia*.

########## Etymology.

The name is derived from the locality Datong County where the new species was discovered.

########## Diagnosis.

Body length 12.3–14.2 mm; antennae composed of 20+20 articles; 10 ocelli on each side arranged in 3 irregular rows, terminal one ocellus comparatively large; To larger than the adjoining ocelli; 2+2 coxosternite teeth and setiform porodonts posterolateral to the lateralmost tooth; posterior angles of all tergites without triangular projections; tarsal articulation well-defined on all legs; legs XII–XV with DaC, leg XV with posterior accessory claw; coxal pores 4–7, round, arranged in one row; female gonopods with 2+2 moderately large, coniform spurs; claw of the third article simple, with a small triangular protuberance on basal ventral side; male gonopods short and small.

########## Description.

Holotype (♀), body 14.2 mm long, cephalic plate width 1.54 mm, length 1.54 mm.

*Colour* red-brown, with a distinct, darker, axial stripe on cephalic plate and tergites. Legs pale yellow-brown. Sternite yellow-brown with distal part brown with reddish hue.

*Antennae* tapering, ca. 4.3 mm long, reaching the anterior part of T V, composed of 20 elongate articles (Figure 1A). Basal article to the seventh article wider than long, following articles elongate, distal article markedly longer than wide, up to 2.2 times as long as wide. Abundant setae on the antennal surface.

*Ocelli area*: ten on each side, dark, arranged in three broken rows; posterior ocellus slightly larger than posterosuperior ocellus and other seriated ocelli. To slightly larger than nearest ocellus, rounded.

*Cephalic plate*: breath/length ratio 1.0 (1.54 mm); smooth, longer setae scattered along the entire surface sparsely and the marginal ridge of the cephalic plate. Transverse suture distinct, lateral marginal ridge discontinuous, posterior margin continuous, slightly concave (Figure 2 A).

*Coxosternite*: dental margin slightly concave, with 2+2 slightly acute teeth and setiform porodonts separated from the lateral tooth laterally, median diastema U-shaped; shoulders of coxosternite strongly sloping, as in Figure 2C. Scattered short setae on the anterior ventral side of coxosternite, longer and stronger setae near the porodonts.

*Tergites* almost smooth. The anterior part of T I is approx. the same width as cephalic plate and T III; T I and T III approximately the same width. Posterior angles of all tergites rounded without triangular projections. Posterior margin of TI straight; posterior margin of TT III, V, VIII, X, XII, and XIV concave; posterior margin of TVII convex; posterior margin of intermediate T straight; TT VI–XIV bordered laterally only (Figure 2A). Short to long setae along the lateral margin and anterior and posterior angles of each tergite.

*Sternites*: posterior side of sternites narrower than anterior, generally trapezoidal, smooth; SS XIII–XV with miniscule setae scattered sparsely over the surface; genital sternite more densely setose, as in Figure 2E; four to five pairs of short to long fine setae along anterior lateral borders and posterior borders of sternites; several fine setae along posterior margins of SS I– XII.

*Legs*: tarsal articulation of all legs distinct. Legs XIV and XV incrassate, without visible modification. Length of legs XV: F = 0.85 mm, Ti = 1.00 mm, Ts I = 0.77 mm, Ts II = 0.54 mm. Legs XII– XV with DaC. All legs with fairly long curved claws; legs I– XIV with anterior and posterior accessory spurs, anterior accessory spur moderately long and slender, posterior accessory spur slightly more robust; the anterior accessory spines form relatively small angles with the main claw, the posterior accessory spines form relatively large angles with the main claw; posterior accessory spines on legs XIV. Numerous glandular pores scattered on the surface of prefemur, femur, tibia, tarsus of legs XIV and XIV; short to long comparatively long setae scattered very sparsely over the surface of all segments of legs I– XIII, more setae scattered on the whole surface of tarsus, slightly thick setae arranged in two rows on the ventral side of tarsus. Plectrotaxy as presented in Table 2.

*Coxal pores* present on legs XII–XV, rounded and separated by distance 0.2–2.5 times greater than their own diameter; inner pores smaller than neighbouring ones; formula 4655 and 5575. Coxal pores 4654 and 4554 in male. Coxal pore field set in a relatively shallow groove, the coxal pore-field fringe with prominence. Prominence with short to moderately long setae sparsely scattered over the surface.

*Female posterior segment* S XV generally trapeziform, straight posteromedially; sternite of genital segment wider than long with posterior margin moderately concave between condyles of gonopods, except for a small, median bulge; distal part lightly sclerotised; short to long setae scattered over the surface of genital segment and lateral margins. Basal article of gonopod bearing 22–25 setae, with two blunt spurs of approximately equal size at distal end of slender, elongate process and three long spines on dorsolateral side; second article of gonopod with 5-6 setae and five long curved spines on dorsolateral side; third article with two setae (Figure 2E). Claw undivided, bearing a small triangular protuberance on ventral side (Figure 2D).

*Male posterior segment* (Figure 2F) S XV subtrapeziform, long setae scattered sparsely over its surface. Sternite of genital segment obviously smaller than the female, well sclerotized; posterior margin quite deeply concave between the gonopods, no bulge medially; gonopods short, appearing as a hemispherical bulge, one segmented, with three setae. Male leg XV not modified.

**Table 2.** Lithobius (Ezembius) datongensis sp. n.: leg plectrotaxy; letters in brackets indicate variable spines.

legs	ventral					dorsal				
	C	Tr	P	F	Ti	C	Tr	P	F	Ti
1			mp	(a)mp	am			ap	a(p)	ap
2–9			amp	amp	am			ap	ap	ap
10			amp	amp	am			ap	ap	ap
11			amp	amp	am			a(m)p	ap	ap
12–14		m	amp	amp	am	a		amp	p	p
15		m	amp	am	a	a		amp	p	

########## Variations.

Body length 12.3–14.2 mm; 9–10 ocelli; coxal pores 4655, 5575, or 5544 in female, 4654 and 4554 in male.

########## Remarks.

The new species can be easily distinguished from the other species of the subgenus of *Ezembius* of China except *Ezembiusanabilineatus* by the apical claw of female gonopods simple with a small subtriangular protuberance on the ventral side. It differs from *E.anabilineatus* in many aspects, such as a larger body, fewer antennal articles (20+20, vs. 23+23 in *E.anabilineatus*), more ocelli, a DaC spine on legs XII–XV (only on legs XIV and XV in *E.anabilineatus*), and a posterior accessory spur present on legs XV present. It differs from Lithobius (Ezembius) longibasitarsus sp. n. by having posterior accessory spur on XV legs, fewer ocelli (10 versus up to 14 in *E.longibarsitarsus*) and different plectrotaxy (VmTr absent on legs XII and XIII vs. present).

######### Key to species of the subgenus Ezembius in China

**Table:** 

1	Posterior angles of tergites with triangular projections	**2**
–	Posterior angles of tergites rounded, without projections	**3**
2	Posterior angles of TT VII, IX, XI, XIII with triangular projections	**L. (E.) kiayiensis Wang, 1959**
–	Posterior angles of TT XIV with slightly triangular projections	**L. (E.) sulcipes Attems, 1927**
3	At most four ocelli on each side of cephalic plate	**L. (E.) parvicornis (Porat, 1893)**
–	At least five ocelli on each side of cephalic plate	**4**
4	Cephalic plate with scattered, rough punctae and tergites with distinct punctae	**L. (E.) rhysus Attems, 1934**
–	Cephalic plate and tergites without any punctae	**5**
5	All ocelli subequal in size	**6**
–	All ocelli not subequal in size	**7**
6	Six ocelli on each side of cephalic plate	**L. (E.) sulcifemoralis Takakuwa & Takashima, 1949**
–	Eight to twelve ocelli on each side of cephalic plate	**L. (E.) sibiricus Gerstfeldt, 1858**
7	Posterior ocellus small	**L. (E.) lineatus Takakuwa, 1939**
–	Posterior ocellus large	**8**
8	The terminal two ocelli comparatively large	**9**
–	The terminal one ocellus comparatively large	**12**
9	Ocelli arranged in two rows	**L. (E.) laevidentata Pei, Ma, Hou, Zhu & Gai, 2015**
–	Ocelli arranged in three rows	**10**
10	3+3 coxosternal teeth	**L. (E.) multispinipes Pei, Lu, Liu, Hou, Ma & Zapparoli, 2016**
–	2+2 coxosternal teeth	**11**
11	Tömösváry’s organ larger than the adjoining ocellus	**L. (E.) bilineatus Pei, Ma, Zhu & Gai, 2014**
–	Tömösváry’s organ smaller than the adjoining ocellus	**L. (E.) anabilineatus Ma, Pei, Hou, Zhu & Gai, 2015**
12	Only five ocelli on each side of cephalic plate	**L. (E.) chekianus Chamberlin & Wang, 1952**
–	At least six ocelli on each side of cephalic plate	**13**
13	Tömösváry’s organ smaller than the adjoining ocellus.	**14**
–	Tömösváry’s organ larger than the adjoining ocellus	**16**
14	Ocelli arranged in three rows	**L. (E.) longibasitarsus sp. n.**
–	Ocelli arranged in two rows	**15**
15	First article of female gonopods with 3+3 spurs	**L. (E.) insolitus Eason, 1993**
–	First article of female gonopods with 2+2 or 2+3 spurs	**L. (E.) irregularis Takakuwa & Takashima, 1949**
16	First article of female gonopods with 1 + 1 spurs	**L. (E.) gantoensis Takakuwa & Takashima, 1949**
–	First article of female gonopods with more than 1+1 spurs	**17**
17	First article of female gonopods with 3+3 or 4+4 spurs	**18**
–	First article of female gonopods with 2+2 or 2+3 spurs	**19**
18	Terminal claw of female gonopods simple without a small subtriangular tooth on inner margin	**L. (E.) mandschreiensis Takakuwa, 1939**
–	Terminal claw of female gonopods simple with a small subtriangular tooth on inner margin	**L. (E.) bidens Takakuwa, 1939**
19	Terminal claw of female gonopods bipartite	**L. (E.) anasulcifemoralis Ma, Pei, Wu & Gai, 2013**
–	Terminal claw of female gonopods not bipartite	**19**
20	Terminal claw of female gonopods tridentate	**L. (E.) zhui Pei, Ma, Shi, Wu & Gai, 2011**
–	Terminal claw of female gonopods simple	**20**
21	Terminal claw of female gonopods simple without a small subtriangular teeth on inner margin	**L. (E.) giganteus Sseliwanoff, 1881**
–	Terminal claw of female gonopods simple with a small subtriangular teeth on inner margin	**L. (E.) datongensis sp. n.**

######### Molecular analysis

***Sequence characterisation*.** Alignment of the PCR fragment sequences from COI showed that in the 613 bp there were 271 variable sites and 258 parsimony informative characters. The base composition of the fragments showed a strong bias of A + T (29.0+32.3). The results of the substitution saturation test showed that the index of substitution saturation 0.2562 (*Iss*) is significantly lower than the critical value of the index of substitution saturation 0.7345 (*Iss. c*).

***Genetic distance*.** Calculation of the distances (Table 3) between different species showed that they ranged from 16.97% (Lithobius (Ezembius) giganteus/*Lithobiusholsti*) to 26.26% (Lithobius (L.) forficatus/*Lamyctesinermipes*) with an average genetic distance of 20.32%. The five sequences of Lithobius (Ezembius) longibasitarsus sp. n. are identical. There is only one nucleotide change in Lithobius (Ezembius) datongensis sp. n. Uncorrected p-distances to the outgroup ranges from 16.64% to 21.70%.

**Figure 3.**
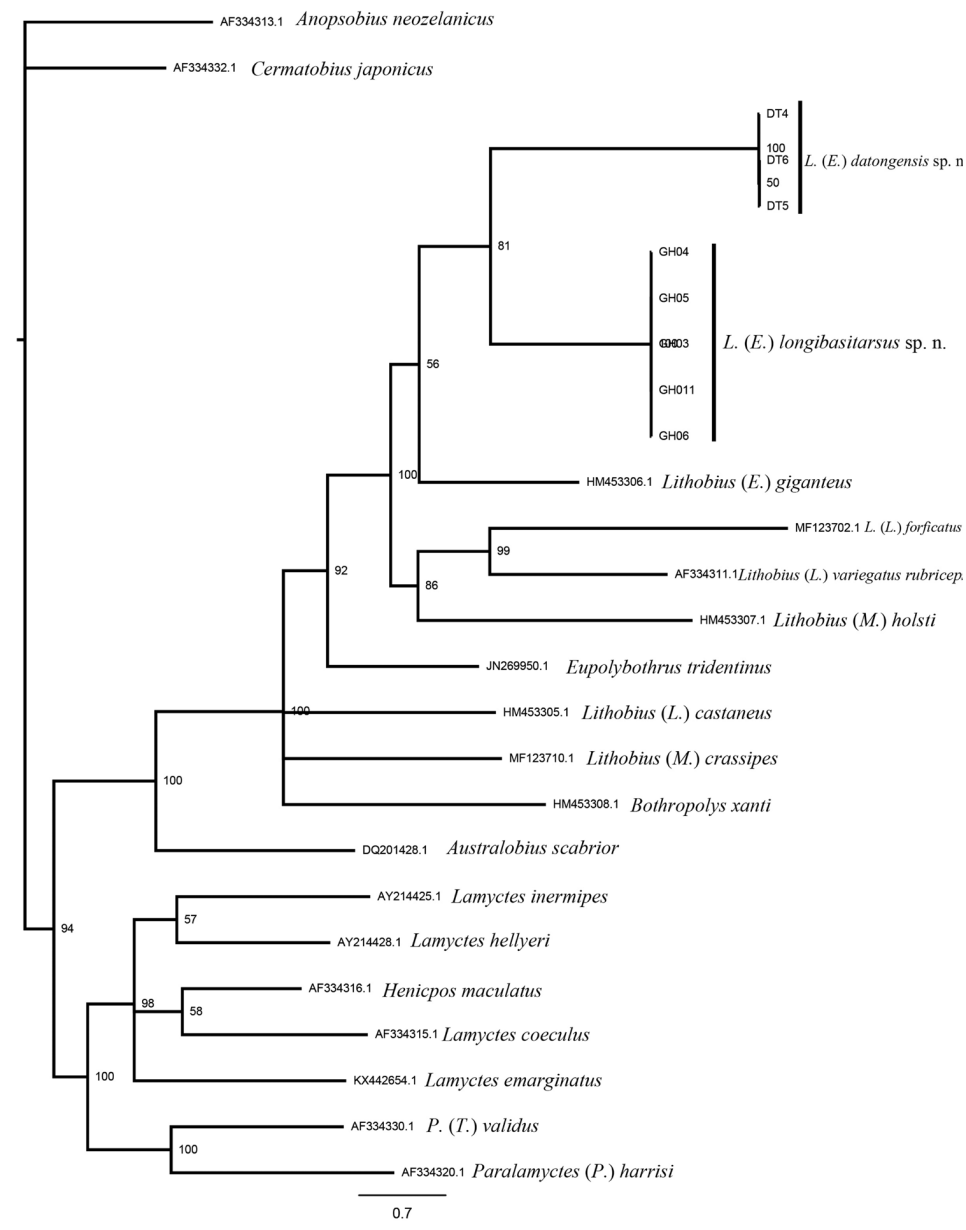
Bayesian tree for the 26 sequences of Lithobiomorpha based on COI sequences. The Bayesian posterior probabilities from Bayesian analyses are presented above the main branches. The scale bar represents substitutions per site.

***Phylogenetic relationship*.** Bayesian inference (BI) analysis (Figure 3) reveal that Lithobiomorpha shows a split between Lithobiidae and Henicopidae with posterior probabilities 94%. The monophyly of *Ezembius* is supported with bootstrap values of 56%. The monophyly of *Paralamyctes* is supported by the COI data, with posterior probabilities of 100%. The genus *Australobius* was placed in the basal position as the sister to the rest of Lithobiidae, which includes the two subfamilies Lithobiinae and Ethopolyinae.

**Table 3.** Estimates of evolutionary divergence between sequences.

	1	2	3	4	5	6	7	8	9	10	11	12	13	14	15	16	17	18	19	20	21	22	23	24	25
1. DT4																									
2. DT6	0.0016																								
3. DT5	0.0016	0.0000																							
4. GH04	0.1794	0.1794	0.1794																						
5. GH05	0.1794	0.1794	0.1794	0.0000																					
6. GH03	0.1794	0.1794	0.1794	0.0000	0.0000																				
7. GH011	0.1794	0.1794	0.1794	0.0000	0.0000	0.0000																			
8. GH06	0.1794	0.1794	0.1794	0.0000	0.0000	0.0000	0.0000																		
9. MF123702.1	0.1990	0.1974	0.1974	0.2055	0.2055	0.2055	0.2055	0.2055																	
10. HM453305.1	0.1876	0.1876	0.1876	0.1811	0.1811	0.1811	0.1811	0.1811	0.2039																
11. MF123710.1	0.1876	0.1892	0.1892	0.2202	0.2202	0.2202	0.2202	0.2202	0.2153	0.1811															
12. HM453306.1	0.1958	0.1941	0.1941	0.1794	0.1794	0.1794	0.1794	0.1794	0.2088	0.1794	0.1941														
13. HM453307.1	0.2104	0.2104	0.2104	0.2055	0.2055	0.2055	0.2055	0.2055	0.2235	0.1713	0.1974	0.1697													
14. AF334311.1	0.2072	0.2072	0.2072	0.1925	0.1925	0.1925	0.1925	0.1925	0.2055	0.2007	0.2202	0.1794	0.1909												
15. JN269950.1	0.1974	0.1974	0.1974	0.1990	0.1990	0.1990	0.1990	0.1990	0.1990	0.1697	0.1843	0.1582	0.1860	0.1778											
16. HM453308.1	0.2219	0.2202	0.2202	0.2039	0.2039	0.2039	0.2039	0.2039	0.2153	0.1925	0.1958	0.1990	0.2023	0.2007	0.1827										
17. AY214425.1	0.2545	0.2561	0.2561	0.2496	0.2496	0.2496	0.2496	0.2496	0.2626	0.2235	0.2333	0.2202	0.2333	0.2577	0.2251	0.2365									
18. AF334316.1	0.2284	0.2268	0.2268	0.2300	0.2300	0.2300	0.2300	0.2300	0.2398	0.2088	0.2251	0.2251	0.2480	0.2545	0.2202	0.2349	0.1599								
19. AY214428.1	0.2512	0.2512	0.2512	0.2349	0.2349	0.2349	0.2349	0.2349	0.2365	0.2170	0.2153	0.2300	0.2186	0.2349	0.2072	0.2251	0.1746	0.1713							
20.AF334315.1	0.2121	0.2137	0.2137	0.2365	0.2365	0.2365	0.2365	0.2365	0.2284	0.2088	0.2039	0.2055	0.2235	0.2414	0.2121	0.2268	0.1794	0.1697	0.1729						
21. KX442654.1	0.2382	0.2398	0.2398	0.2300	0.2300	0.2300	0.2300	0.2300	0.2316	0.2219	0.2039	0.2316	0.2398	0.2414	0.2186	0.2349	0.1909	0.1843	0.1876	0.1876					
22. AF334330.1	0.2398	0.2414	0.2414	0.2398	0.2398	0.2398	0.2398	0.2398	0.2219	0.2382	0.2186	0.2496	0.2414	0.2463	0.2170	0.2594	0.2202	0.2023	0.1925	0.1958	0.1892				
23. AF334332.1	0.2529	0.2512	0.2512	0.2431	0.2431	0.2431	0.2431	0.2431	0.2431	0.2349	0.2251	0.2186	0.2349	0.2349	0.1925	0.2072	0.2055	0.1778	0.1876	0.1990	0.2072	0.1925			
24. AF334313.1	0.2284	0.2284	0.2284	0.2170	0.2170	0.2170	0.2170	0.2170	0.2121	0.1892	0.2039	0.2170	0.1892	0.2219	0.1762	0.2121	0.2055	0.1958	0.1664	0.1827	0.1925	0.1941	0.1860		
25. AF334320.1	0.2316	0.2316	0.2316	0.2219	0.2219	0.2219	0.2219	0.2219	0.2235	0.2104	0.2480	0.2251	0.2284	0.2300	0.2251	0.2300	0.2219	0.1974	0.1941	0.2153	0.2072	0.1990	0.2039	0.1794	
26. DQ201428.1	0.2202	0.2186	0.2186	0.2235	0.2235	0.2235	0.2235	0.2235	0.2186	0.1958	0.1974	0.1974	0.2202	0.2007	0.1860	0.2055	0.1876	0.1925	0.1860	0.1974	0.1925	0.2153	0.1860	0.1876	0.1892

## Discussion

Both molecular analysis (Figure 3) and morphology support that the two new species belong to the subgenus Ezembius. The subgenus Ezembius (sensu Eason 1986, 1992) has an Asiatic distribution which extends from the Urals across Siberia and central Asia to China, Japan, and Alaska, southwards into the northern Indian subcontinent and the northern part of the oriental region, and the southwest extremity to Israel and neighbouring areas (Eason 1992, Negrea 2005).

The intraspecific distance (less than 1%), is significantly less than interspecific distance (more than 10%), so the COI can be used for species identification. The intraspecific genetic polymorphism of Lithobius (E.) longibasitarsus sp. n. and Lithobius (E.) datongensis sp. n.) is very low and could indicate weak migration and diffusion capacity with strong natural selection. Assuring the monophyly and interrelationships of the many genera and subgenera belong to Lithobiidae needs further intensive study including more diverse sampling and molecular evidence, the direction of our future effects.

## Supplementary Material

XML Treatment for Lithobius (Ezembius) longibasitarsus

XML Treatment for Lithobius (Ezembius) datongensis

## Appendix 1

**Table:** Species used for CO1 sequence analysis, sequence references, GenBank accession numbers, voucher, and locality. ZSM = Zoologische Staatssammlung München, Germany; AM KS = Australian Museum; MCZ = Museum of Comparative Zoology, Harvard University; SMNG = Senckenberg Museum of Natural History.

Taxa	Sequence reference	GenBank No.	Voucher	Locality
Lithobiidae				
Lithobiinae				
Lithobius (Monotarsobius) crassipes	Voigtländer et al. 2017	MF123710.1	SMNG VNR 17281-1	France,
Lithobius (L.) forficatus	Voigtländer et al. 2017	MF123702	SMNG VNR 17150-2	Germany
Lithobius (L.) variegatusrubriceps	Murienne et al. 2010	AF334311	MCZ DNA100283	Spain
Lithobius (L.) castaneus	Murienne et al. 2010	HM453305	MCZ DNA103939	Italy
Lithobius (Ezembius) giganteus	Murienne et al. 2010	HM453306	MCZ DNA101089	Kazakhstan
Lithobius (E.) longibasitarsus sp. n.	this paper	MH05602	GH04	China
Lithobius (E.) longibasitarsus sp. n.	this paper	MH05603	GH05	China
Lithobius (E.) longibasitarsus sp. n.	this paper	MH05604	GH03	China
Lithobius (E.) longibasitarsus sp. n.	this paper	MH05605	GH011	China
Lithobius (E.) longibasitarsus sp. n.	this paper	MH05606	GH06	China
Lithobius (E.) datongensis sp. n.	this paper	MH05607	DT4	China
Lithobius (E.) datongensis sp. n.	this paper	MH05608	DT6	China
Lithobius (E.) datongensis sp. n.	this paper	MH05609	DT5	China
Lithobius (M.) holsti	Murienne et al. 2010	HM453307	MCZ DNA102106	Japan
* Australobius scabrior *	Giribet and Edgecombe 2006	DQ201428		Australia
Ethopolyinae				
* Eupolybothrus tridentinus *	Stoev et al. 2013	JN269950.1	BC ZSM MYR 00430	Croatia
* Bothropolys xanti *	Murienne et al. 2010	HM453308	Bmultide	USA
Henicopidae				
Anopsobiinae				
* Anopsobius neozelanicus *	Edgecombe et al. 2002	AF334313.1	AM KS 57958	New Zealand
Henicopinae				
Henicopini				
* Henicops maculatus *	Edgecombe and Giribet 2003	AF334316.1	AM KS 57962	Australia
* Lamyctes coeculus *	Edgecombe and Giribet 2003	AF334315.1	MCZ DNA100288	Australia
* Lamyctes emarginatus *	Voigtländer et al. 2017	KX442654.1	ZSM-JSP120527-016	Germany
* Lamyctes inermipes *	Edgecombe and Giribet 2003	AY214425.1	MCZ DNA100478	Argentina
* Lamyctes hellyeri *	Edgecombe and Giribet 2003	AY214428.1	MCZ DNA100639	Australia
Paralamyctes (P.) harrisi	Edgecombe et al. 2002	AF334320	AM KS 57971	New Zealand
P. (Thingathinga) validus	Edgecombe et al. 2002	AF334330	AM KS 57969	New Zealand
Zygethobiini				
* Cermatobius japonicus *	Edgecombe et al. 2002	AF334332	MCZ 28612	Japan
